# Comparative Study of Vaginal Misoprostol Tablet Versus Dinoprostone Insert in Induction of Labor: A Prospective Interventional Analysis

**DOI:** 10.7759/cureus.80026

**Published:** 2025-03-04

**Authors:** Vaishnavi Unni, Subhashchandra R Mudanur, Rajasri G Yaliwal, Shreedevi Kori

**Affiliations:** 1 Department of Obstetrics and Gynaecology, Shri B. M. Patil Medical College and Research Centre, BLDE (Deemed to be University), Vijayapura, IND; 2 Department of Obstetrics and Gynaecology, Shri B. M. Patil Medical College and Research Centre, Vijayapura, IND

**Keywords:** dinoprostone, labor induction, misoprostol, prostaglandin, vaginal insert

## Abstract

Introduction

Induction of labor (IOL) is an essential obstetric procedure for maternal and fetal safety when continuing pregnancy is risky. Prostaglandin analogs like misoprostol (Prosaglandin E1 (PGE1)) and Dinoprostone (Prostaglandin E2 (PGE2)) are commonly used for cervical ripening and labor induction. This study compares the efficacy and safety of vaginal tablet misoprostol versus Dinoprostone insert in the IOL.

Materials and methods

This prospective, interventional study was conducted at BLDE University, Vijayapura, from April 2023 to February 2025, involving 106 pregnant women at term. Participants were divided into two groups: Group 1 received a 10 mg Dinoprostone vaginal insert, while Group 2 received 25 mcg vaginal misoprostol every four hours. Key outcomes included vaginal delivery rates, cesarean section rates, and maternal and neonatal complications. Data were analyzed using SPSS.

Results

Vaginal delivery occurred in 39 (73.58%) of the Dinoprostone group compared to 27 (50.94%) in the misoprostol group (p=0.02). The mean induction-to-delivery interval was significantly shorter in the misoprostol group (15.2 ± 4.9 hours vs. 18.3 ± 4.29 hours, p<0.001). Maternal complications, including postpartum hemorrhage (PPH), were more common in the misoprostol group 13 (24.5%). Neonatal complications, such as neonatal intensive care unit (NICU) admissions and lower Apgar scores, were also significantly higher in the misoprostol group.

Conclusion

Both misoprostol and Dinoprostone are effective for labor induction, but misoprostol shortens the induction-to-delivery interval at the cost of increased cesarean rates and fetal complications. Dinoprostone, while slower, shows better fetal outcomes and fewer complications, making it a preferable option for high-risk pregnancies. Tailoring the choice of induction agent based on patient-specific factors is essential.

## Introduction

Induction of labor (IOL) is a critical obstetric intervention undertaken when the risks of continuing pregnancy outweigh the benefits for the mother or fetus [1]. Globally, the rate of labor induction has risen significantly, with recent statistics showing induction rates as high as 34% in the UK, 23% in the USA, and 25-35% in Australia [2]. A recent study estimates that the labor induction rate in India is around 42.3% [3]. The primary goal of IOL is to achieve a successful vaginal delivery while minimizing complications for both the mother and newborn [4]. Among the various pharmacological methods used for cervical ripening and labor induction, prostaglandin analogs, most commonly used are Dinoprostone and misoprostol [5,6].

Misoprostol (Prostaglandin E1 (PGE1)) has become a key agent in obstetric practice due to its efficacy in promoting cervical ripening and uterine contractions [7]. It is administered orally, vaginally, or sublingually, offering flexibility in dosing and ease of storage without refrigeration [8]. In contrast, Dinoprostone (Prostaglandin E2 PGE2) is a naturally occurring prostaglandin that requires refrigeration and is typically administered as an intracervical gel or vaginal insert [9,10]. Both agents act by stimulating myometrial contractions and softening the cervix, but they differ in their pharmacokinetics, safety profiles, and effectiveness [11].

While some studies suggest that misoprostol shortens induction-to-delivery time and increases the rate of vaginal delivery, but also causes uterine hyperstimulation and abnormalities in fetal heart rate [4,5,12]. Conversely, Dinoprostone is often linked to a longer induction process but with a more controlled and predictable labor progression [4,5]. Recent meta-analyses have highlighted that misoprostol may cause lesser maternal complications compared to Dinoprostone, though perinatal outcomes remain comparable [2].

Despite extensive research, there remains no universal consensus on the ideal agent for labor induction. Factors such as maternal parity, gestational age, cervical status, and institutional protocols influence the choice of prostaglandin used [13]. This study aims to provide a comparative analysis of efficacy and safety of Dinoprostone pessary vs vaginal tablet misoprostol in IOL, evaluating maternal and fetal outcomes. By analyzing these two commonly used methods, we seek to contribute to the ongoing discourse on optimizing induction protocols for improved perinatal care.

## Materials and methods

This prospective interventional study was conducted in the Department of Obstetrics and Gynecology at Shri B. M. Patil Medical College Hospital and Research Centre, over a period from April 2023 to February 2025. The study involved patients admitted for induction of the labor who met the inclusion criteria. Singleton pregnant women at term (>37 weeks) with a cephalic presentation fetus with no signs of labor before induction were included in the study. Exclusion criteria consisted of patients contraindicated for vaginal delivery, such as placenta previa, vasa previa, prior surgical procedures on the uterine body (including previous lower segment cesarean section, myomectomy, and cases of prior uterine rupture), invasive cervical cancer, active genital herpes infection, malpresentation, malposition, and pre-labor rupture of membranes (PROM). Eligible patients were randomized into two groups and were blinded to the assigned intervention. Institutional Ethics Committee, BLDE, Vijayapura approved the study (Approval number: IEC/896/2022-23).

Patients in Group 1 received a 10 mg Dinoprostone vaginal insert, reassessed every 12 hours, and removed either after 24 hours or when the active phase of labor commenced. Group 2 received vaginal misoprostol (25 mcg) inserted high in the posterior fornix and repeated every four hours as required. In cases where spontaneous labor did not occur, oxytocin augmentation and amniotomy were performed at 4-6 cm cervical dilation. Induction failure was defined as an absence of active labor after 12 hours of oxytocin infusion. Arrested labor was determined if there was no cervical dilation progression for four hours or no fetal descent or rotation for two hours during the second stage of labor. The study compared the two interventions in terms of vaginal delivery rates, cesarean section rates, and vacuum-assisted births, as well as maternal and neonatal outcomes, including meconium-stained liquor, postpartum hemorrhage (PPH), and neonatal distress.

**Figure 1:**
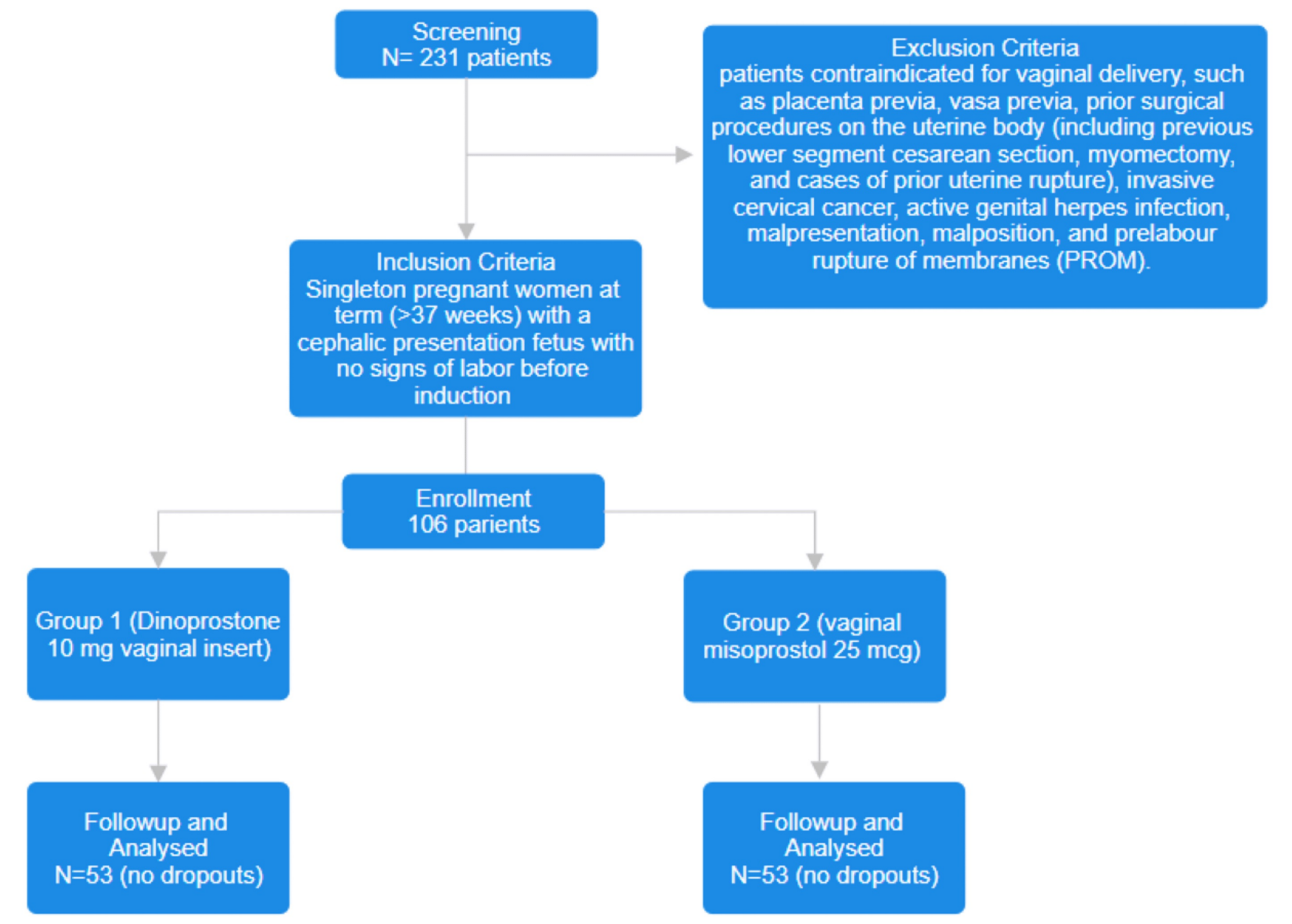
CONSORT Flow Diagram CONSORT: Consolidated Standards of Reporting Trials

The required sample size for the study was calculated at 106 patients, with 53 participants in each group (Figure 1). Data were analyzed using SPSS version 26 (IBM, Armonk, USA). Continuous variables were expressed as Mean±SD and compared using an independent t-test. Categorical variables were analyzed using the chi-square test and a p-value of less than 0.05 was considered statistically significant.

## Results

The study included 106 participants, with 53 in each group receiving either Dinoprostone or vaginal misoprostol for labor induction. Maternal age distribution was similar between the groups (p=0.695), with the majority aged 21-30 years. Parity was also comparable, with 26 (41.9%) primiparous in the Dinoprostone group and 28 (52.8%) in the vaginal misoprostol group (p=0.698). However, the mean gestational age was slightly higher in the Dinoprostone group (39.5 ± 1.15 weeks) compared to the vaginal misoprostol group (39 ± 1.13 weeks, p=0.029) (Table 1).

**Table 1:** Clinical Characteristics The data has been represented as N (%) or Mean±SD. Also, p-value is considered significant at p<0.05.

Characteristic		Dinoprostone (n=53)	Vaginal Misoprostol (n=53)	Statistical Value	p-value
Maternal Age (Years)	≤ 20	10 (18.9%)	11 (20.8%)	Chi-square value: 0.726	0.695
	21-30	39 (73.6%)	40 (75.5%)		
	≥ 31	4 (7.5%)	2 (3.8%)		
Parity	Primiparous	26 (41.9%)	28 (52.8%)	Chi-square value: 0.151	0.698
	Multiparous	27 (50.9%)	25 (47.2%)		
Mean Gestational Age (Weeks)		39.5 ± 1.15	39 ± 1.13	t-value: 2.053	0.029

Normal vaginal delivery (NVD) rates were 39 (73.58%) for the Dinoprostone group versus 27 (50.94%) for the vaginal misoprostol group (p=0.043). The cesarean section rate was higher in the misoprostol group 25 (47.17%) compared to Dinoprostone 14 (26.42%)(p=0.043). The mean induction-to-delivery interval was shorter for misoprostol (15.2 ± 4.9 hours) than for Dinoprostone (18.3 ± 4.29 hours, p<0.001). Final Bishop’s scores were similar (p=0.64) (Table 2).

**Table 2:** Labor Outcomes The data has been represented as N (%) or Mean±SD. Also, p-value is considered significant at p<0.05. LSCS: Lower segment cesarean section

Outcome		Dinoprostone (n=53)	Vaginal Misoprostol (n=53)	Statistical Value	p-value
Mode of Delivery	Vaginal delivery	39 (73.58%)	27 (50.94%)	Chi-square value: 6.284	0.043
	LSCS	14 (26.42%)	25 (47.17%)		
	Assisted vaginal delivery	0 (0%)	1 (1.89%)		
Mean Induction-to-Delivery Interval (Hours)		18.3 ± 4.29	15.2 ± 4.9	t-value: 4.523	<0.001
Final Bishop’s Score		10.3 ± 2.66	10 ± 2.41	t-value: 0.141	0.64

Meconium-stained liquor was more common in the vaginal misoprostol group 13 (24.5%) than in the Dinoprostone group 6 (11.3%)(p=0.076). Neonatal intensive care unit (NICU) admissions were significantly higher in the misoprostol group 25 (47.17%) compared to the Dinoprostone group 9 (16.98%)(p<0.001). Apgar scores <8 at 1 minute were also more frequent in the misoprostol group 22 (41.5%) compared to the Dinoprostone group 9 (17%, p=0.006). Uterine tachysystole was more prevalent in the misoprostol group 10 (18.87%) than in the Dinoprostone group 3 (5.66%)(p=0.038) (Table 3).

**Table 3:** Maternal and Fetal Complications The data has been represented as N (%) and p-value is considered significant at p<0.05. NICU: Neonatal intensive care unit

Complication	Dinoprostone (n=53)	Vaginal Misoprostol (n=53)	Chi-square Value	p-value
Meconium-Stained Liquor	6 (11.3%)	13 (24.5%)	3.142	0.076
NICU Admission	9 (16.98%)	25 (47.17%)	11.084	<0.001
Apgar Score < 8 at 1 min	9 (17%)	22 (41.5%)	7.704	0.006
Uterine Tachysystole	3 (5.66%)	10 (18.87%)	4.296	0.038

In cesarean section patients, fetal distress was the main reason, affecting 12 (46.15%) in the misoprostol group versus 2 (15.38%) in the Dinoprostone group (p=0.083). Other indications, like failed induction with non-reassuring non stress test (NST) and thick meconium-stained liquor, were similar across groups. Persistent fetal tachycardia occurred only in the Dinoprostone group (Table 4).

**Table 4:** Indications for LSCS The data has been represented as N (%) and p-value is considered significant at p<0.05. LSCS: lower segment cesarean section; NST: non stress test

Indication	Dinoprostone (n=13)	Vaginal Misoprostol (n=26)	Chi-square Value	p-value
Fetal distress	2 (15.38%)	12 (46.15%)	5.860	0.083
Failed induction with non-reassuring NST	1 (7.69%)	3 (11.54%)		
Thick meconium-stained liquor	1 (7.69%)	2 (7.69%)		
Non-reassuring NST	1 (7.69%)	1 (3.85%)		
Persistent tachycardia	1 (7.69%)	0 (0%)		
Other causes	7 (53.85%)	8 (30.77%)		

Maternal outcomes showed a higher rate of PPH in the vaginal misoprostol group 13 (24.5%) compared to the Dinoprostone group 6 (11.3%)(p=0.22). Placental abruption occurred in three (5.7%) of the misoprostol group and two (3.8%) of the Dinoprostone group (p=0.22). Normal maternal outcomes were more frequent in the Dinoprostone group 45 (84.9%) versus the misoprostol group 37 (69.8%)(p=0.22). The primary indication for induction was post-dated pregnancy, at 31 (58.5%) for Dinoprostone and 21 (39.6%) for misoprostol (p=0.131). Other indications showed no major differences between the groups (Table 5).

**Table 5:** Maternal Outcome and Induction Indications The data has been represented as N (%) and p-value is considered significant at p<0.05.

Outcome/Indication		Dinoprostone (n=53)	Vaginal Misoprostol (n=53)	Chi-square Value	p-value
Maternal Outcome	Postpartum hemorrhage (PPH)	6 (11.3%)	13 (24.5%)	3.559	0.22
	Placental abruption	2 (3.8%)	3 (5.7%)		
	Normal outcome	45 (84.9%)	37 (69.8%)		
Indication for Induction	Post-dated pregnancy	31 (58.5%)	21 (39.6%)	7.491	0.131
	Pregnancy-induced hypertension (PIH)	6 (11.3%)	7 (13.2%)		
	Intrauterine growth restriction (IUGR)	0 (0%)	4 (7.5%)		
	Gestational diabetes mellitus	1 (1.9%)	3 (5.7%)		
	Other causes	14 (26.4%)	18 (34.0%)		

## Discussion

IOL is a widely utilized intervention aimed at ensuring timely delivery in cases where continuing pregnancy poses a risk to maternal or fetal health. Among pharmacological agents, Dinoprostone and misoprostol are the most common prostaglandins used for cervical ripening and labor induction.

The present study found that misoprostol significantly reduced the induction-to-delivery interval (15.2 ± 4.9 hours) compared to Dinoprostone (18.3 ± 4.29 hours, p<0.001). This is consistent with findings by Patabendige et al. (2024), who reported that low-dose vaginal misoprostol (≤50 mcg every 4 hours) led to faster labor progression compared to vaginal Dinoprostone [2]. Similarly, Valvi and Airao (2021) showed a shorter labor duration with misoprostol (9.54 hours) than Dinoprostone (13.45 hours), reinforcing its efficacy in accelerating labor [4]. However, despite its faster action, misoprostol was associated with a higher cesarean section rate 25(47.17%) compared to Dinoprostone 14(26.42%), with fetal distress being the primary indication (12 (46.15%) vs. 2 (15.38%))(p=0.083). These findings align with those of Sire et al. (2022), who reported an increased cesarean rate with misoprostol due to abnormal fetal heart rate patterns [12].

Regarding vaginal delivery rates, the present study observed that Dinoprostone led to a higher rate of vaginal delivery 39 (73.58%) compared to misoprostol 27 (50.94%) (p=0.02). This contrasts with Papanikolaou et al. (2004), who reported higher vaginal delivery rates with misoprostol 74 (92.5%) compared to Dinoprostone 72 (86.7%), indicating that misoprostol may be more effective in achieving vaginal birth when labor progresses without complications [11]. Similarly, Valvi and Airao (2021) found that misoprostol resulted in a vaginal delivery rate of 45 (80.35%), compared to 35 (62.5%) with Dinoprostone [4].

Maternal complications, particularly uterine tachysystole, were more frequent in the misoprostol group 10 (18.87%) compared to the Dinoprostone group 3 (5.66%) (p=0.038). These findings align with those of Papanikolaou et al. (2004), who reported that uterine tachysystole was more common in the misoprostol group 10 (12.6%) compared to the Dinoprostone group 3 (3.6%) [11]. Similarly, Valvi and Airao (2021) found that misoprostol had a higher incidence of uterine tachysystole four (7.8%) compared to Dinoprostone one (2.56%), suggesting that misoprostol, while effective, carries a higher risk of uterine hyperstimulation [4]. This is a significant concern as excessive uterine contractions can compromise fetal oxygenation, leading to distress and increased neonatal morbidity [14].

Fetal outcomes in the present study further support the higher incidence of neonatal complications with misoprostol. The study found that NICU admissions were significantly higher in the misoprostol group 25 (47.17%) compared to the Dinoprostone group 9 (16.98%)(p<0.001). Additionally, Apgar scores below 8 at 1 minute were more frequent in the misoprostol group 22 (41.5%) than the Dinoprostone group 9 (17%)(p=0.006). These results align with those of Sire et al. (2022), who found that fetal distress was more frequent in the misoprostol group, leading to higher NICU admissions [12].

The dosages used in this study align with standard clinical guidelines. However, evidence suggests that misoprostol’s safety profile is dose-dependent. Patabendige et al. (2024) emphasized that lower doses of misoprostol (≤25 mcg every 4-6 hours) could achieve similar efficacy while reducing complications [2]. Similarly, Sire et al. (2022) found that higher doses of misoprostol (50 mcg every 6 hours) were associated with increased fetal distress and cesarean rates, reinforcing the need for careful dose titration [12]. Swami and Sonawane (2023) noted that Dinoprostone’s controlled cervical ripening effect may contribute to safer labor progression, leading to better fetal outcomes [15].

Based on these findings, the choice of induction agent should be decided as per individual patient characteristics. Misoprostol may be more suitable for patients requiring a faster labor progression, but its higher risk of hyperstimulation and fetal distress necessitates close monitoring. Conversely, Dinoprostone, despite its longer induction duration, appears safer for fetal outcomes and is preferable for cases with a high risk of fetal compromise. These findings agree with Valvi and Airao (2021) and Patabendige et al. (2024), both of whom recommended that clinicians should weigh the trade-off between induction speed and fetal safety when choosing between these agents [2,4].

While misoprostol effectively reduces labor duration, it is known to cause higher incidence of fetal distress, leading to increased NICU admissions and cesarean deliveries. In contrast, Dinoprostone, though slower in action, provides better fetal outcomes and a higher vaginal delivery rate. The selection of an induction method should be individualized, considering maternal and fetal risk factors, proper dosing strategies, and the need for continuous fetal monitoring to mitigate adverse effects. Further research is warranted to identify optimal induction protocols that balance efficacy with safety, ensuring the best possible maternal and neonatal outcomes.

This study has several limitations. Its single-center design may limit the generalizability of the results. The sample size of 106 participants may not provide enough power to detect smaller differences in less common complications. Although blinding was implemented, the open-label nature of the interventions may introduce bias. Additionally, the study did not include long-term follow-up data on maternal and neonatal health or assess patient satisfaction, which could provide further insights into the outcomes of labor induction.

## Conclusions

This study demonstrates that while misoprostol effectively shortens the induction-to-delivery interval but also causes fetal distress and increased chances of cesarean delivery. In contrast, Dinoprostone causes higher NVD with fewer fetal complications, although labor progression is slower. The choice of an induction agent should be individualized based on maternal and fetal conditions, cervical status, and the need for careful fetal monitoring. Optimizing dosage and administration protocols can help balance efficacy and safety, ensuring better outcomes for both mother and baby.
